# Lithium peroxide crystal clusters as a natural growth feature of discharge products in Li–O_2_ cells

**DOI:** 10.3762/bjnano.4.86

**Published:** 2013-11-15

**Authors:** Tatiana K Zakharchenko, Anna Ya Kozmenkova, Daniil M Itkis, Eugene A Goodilin

**Affiliations:** 1 Department of Materials Science, Moscow State University, Leninskie gory, Moscow 119991, Russia; 2 Department of Chemistry, Moscow State University, Leninskie gory, Moscow 119991, Russia

**Keywords:** lithium–air batteries, lithium peroxide, oxygen reduction reaction

## Abstract

The often observed and still unexplained phenomenon of the growth of lithium peroxide crystal clusters during the discharge of Li–O_2_ cells is likely to happen because of self-assembling Li_2_O_2_ platelets that nucleate homogeneously right after the intermediate formation of superoxide ions by a single-electron oxygen reduction reaction (ORR). This feature limits the rechargeability of Li–O_2_ cells, but at the same time it can be beneficial for both capacity improvement and gain in recharge rate if a proper liquid phase mediator can be found.

## Findings

The idea to utilize oxygen as an oxidizer in rechargeable batteries has been kept in mind for a long time because of the easy availability of O_2_ in ambient air. Alkali metal negative electrodes were always attractive for metal–oxygen (metal–air) batteries as they show record parameters, which originate from the remarkably negative standard electrode potentials. Such cells have already been designed with lithium [1] or sodium [2] anodes and aprotic electrolytes. Unfortunately the practical specific energies are too far from theoretical values and, at the moment, the application of alkali-metal–air rechargeable batteries is impossible because of the very limited cycle life, which primarily arises from the low chemical stability of the electrolytes [3] and the carbon positive electrodes [4]. The oyxgen reduction reaction, which occurs in the cathodes during the discharge of the batteries, leads to the formation of superoxide anions O_2_^−^ that can survive in some aprotic solvents for a certain time [5] and participate in various side reactions. In the case of sodium–oxygen cells superoxide quickly associates with Na^+^ ions and forms well-facetted cubic sodium superoxide (NaO_2_) crystals, which are insoluble in the electrolytes and precipitate onto the electrode [2]. The situation is different for lithium–oxygen cells [6–8], in which LiO_2_ cannot be formed, because it does not exist as a bulk phase at room temperature [9–10]. Instead, all the intermediates have to transform into lithium peroxide (Li_2_O_2_) [11], which always demonstrates a very complex morphology [12] revealing sphere-, torroid- or rozette-like aggregates of plate-like particles. This repeated observation evidences a complex formation mechanism of lithium peroxide.

**Figure 1:**
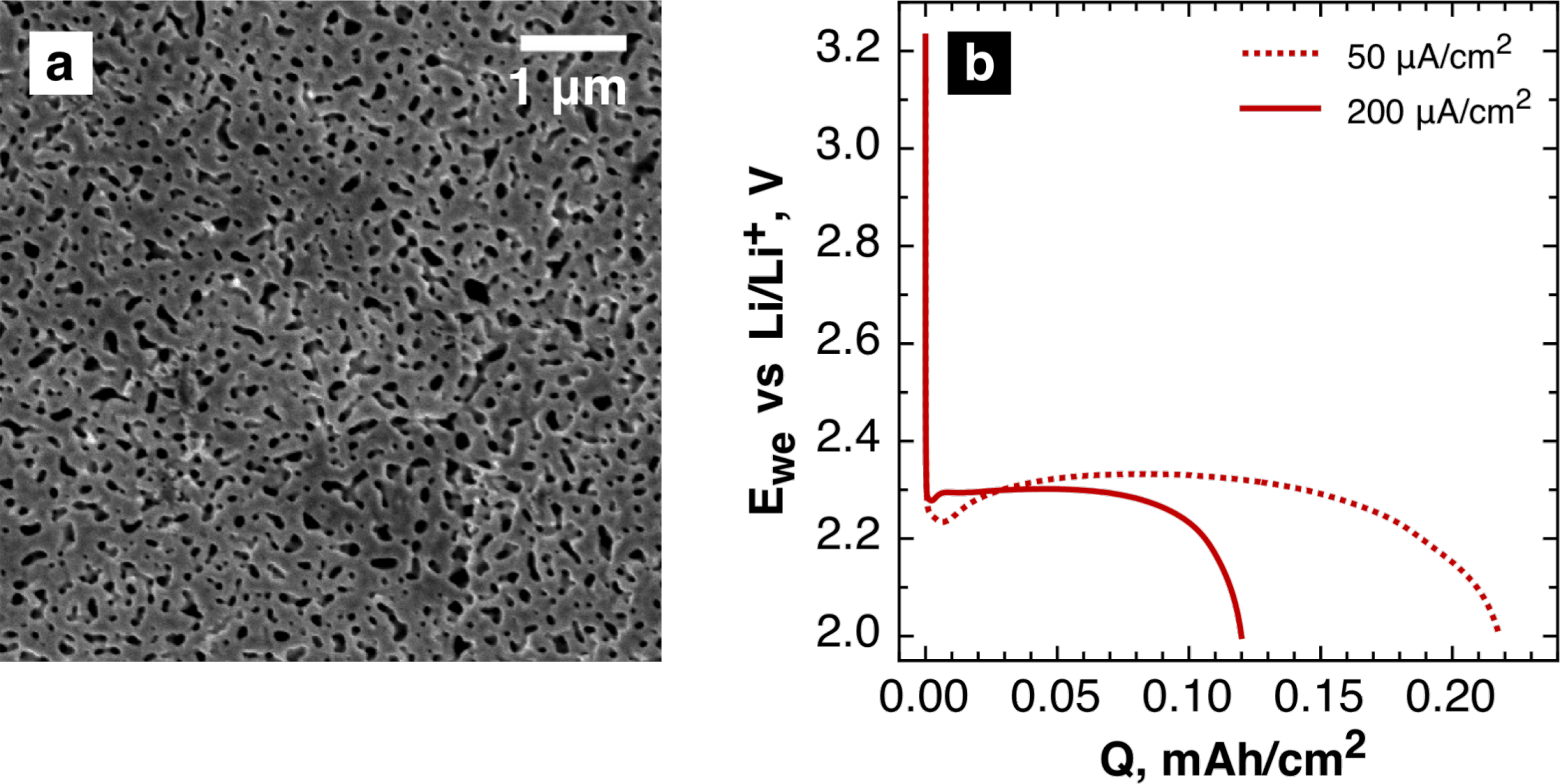
(a) Typical SEM image of the pristine porous gold electrodes. (b) Discharge voltage profiles recorded in a galvanostatic regime at current densities of 50 μA/cm^2^ and 200 μA/cm^2^.

Here we report a new study of Li_2_O_2_ crystals growth upon the discharge of aprotic lithium–oxygen cells. We show that lithium peroxide plate-like crystals are likely to be formed in the liquid electrolyte phase rather than directly on the electrode surface. Li_2_O_2_ particles aggregate to produce finally submicron crystal clusters with different morphologies. To perform all the experiments, we utilized porous gold electrodes with an enhanced surface area and high stability with respect to all redox processes and interaction with peroxide and superoxide species (see Supporting Information File 1 for details). The porous gold electrodes that were utilized as the model electrodes (Figure 1a) were prepared from gold–silver alloy foils (see Supporting Information File 1 for experimental details). The mean pore size was estimated to be about 200 nm (see Figure S1 in Supporting Information File 1). This allowed us to observe changes in the morphology of lithium peroxide that are caused by the varying electrochemical experiment conditions. No side processes were expected for the chosen electrodes [13].

**Figure 2:**
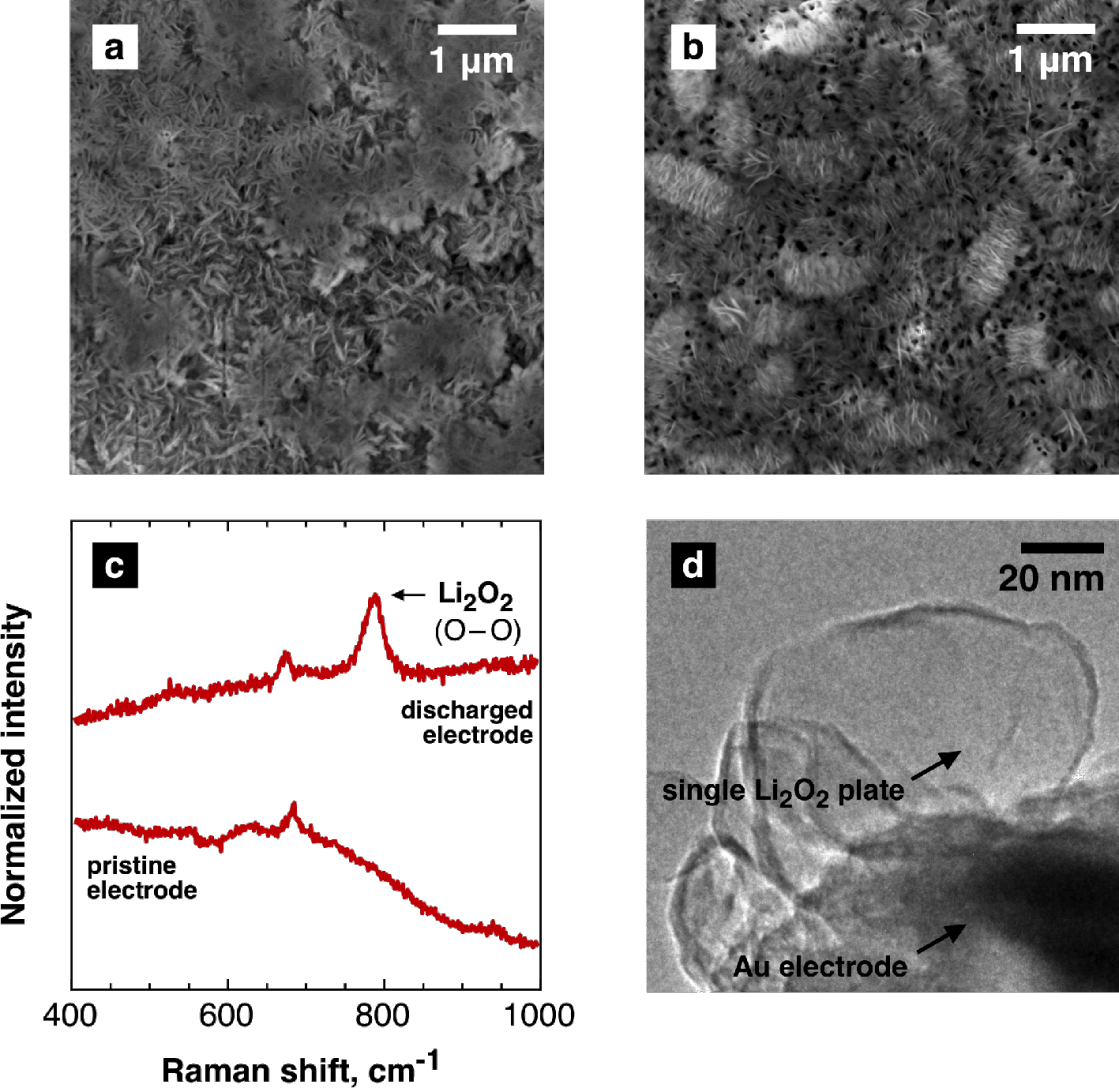
SEM images of the porous gold electrode discharged at 50 μA/cm^2^ (a) and 200 μA/cm^2^ (b). (c) Raman spectra of the pristine and discharged electrodes. (d) TEM image of the porous gold electrode after discharge. Li_2_O_2_ plate edges are deformed by the electron beam.

The galvanostatic discharge of the cells with such electrodes at different current densities (Figure 1b) in an oxygen-saturated 1 M solution of LiTFSI in dry DMSO resulted in the deposition of a porous Li_2_O_2_ layer on the gold surface (Figure 2a,b). The band at 790 cm^−1^ in the Raman spectra of the electrodes after the discharge (Figure 2c) is attributed to O–O stretch vibrations of lithium peroxide [14]. One of the most important experimental conditions is the product generation rate that is being controlled predominantly by the discharge current density. Actually, this parameter determines a supersaturation level, which is required for nucleation and growth of solid phases. We found that the discharge at a lower current density (50 μA/cm^2^) results in more dense Li_2_O_2_ films composed of small building blocks (Figure 2a) while separate stacks of Li_2_O_2_ plate-like crystals grew on the electrode operated at 200 μA/cm^2^ (Figure 2b). The total amount of product formed on the electrodes is higher for low current density, which is in agreement with the area-specific capacities of the cathodes (Figure 1b). Both Li_2_O_2_ film and stacks comprise thin lithium peroxide plates with a diameter varing from 100 to 300 nm that corresponds to previously published data [15]. A typical TEM image of such platelets is presented in Figure 2d, their size of 30–50 nm seems to be natural for as-generated nuclei (embryo crystals) rather than for normally grown anisotropic crystals. The plate-like shape of the crystals can be expected as it is predicted by the Wulff rule [14–15].

The observation of the same building blocks that compose the complex morphologies of Li_2_O_2_ obtained in different electrochemical experiments allows to assume that the formation occurs more or less independently from the generation rate of superoxide ions. In the case of a heterogeneous nucleation of platelets on the surface of the gold electrode, a higher discharge current, and thus a higher concentration of the species being crystallized, would result in a denser layer of lithium peroxide, which is, obviously, opposite to our findings (Figure 1a,b). It seems plausible that the lithium peroxide forms aside of the electrode surface which plays the role of a generator for precursors that are subsequently converted to Li_2_O_2_.

**Figure 3:**
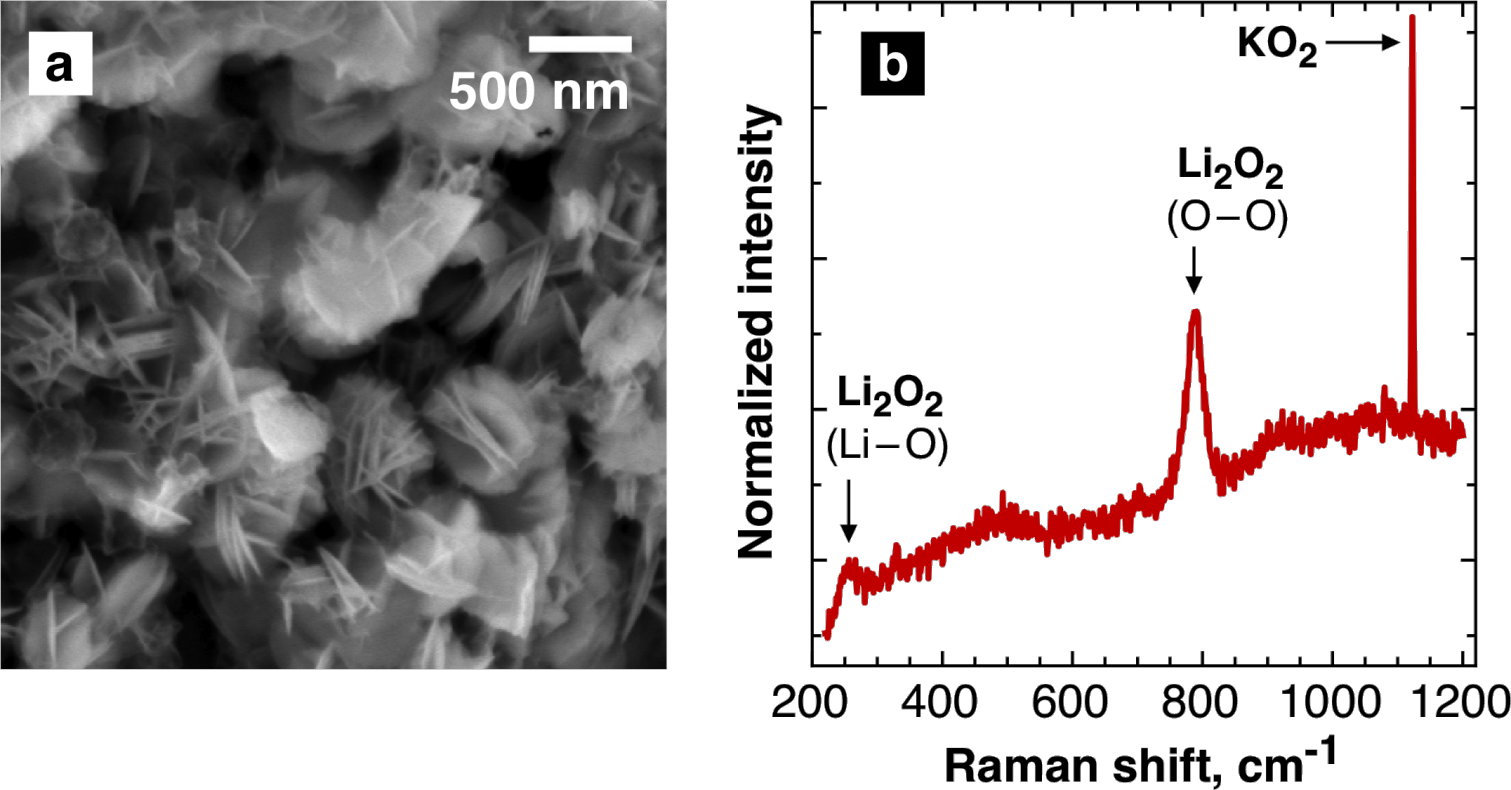
(a) SEM image of the Li_2_O_2_ precipitate obtained by the chemical reaction of KO_2_ with a solution of LiTFSI. (b) Raman spectra of the precipitate.

To find out the most probable way for the generation of such building blocks, we performed a simple experiment purely based on the chemical generation of lithium peroxide in the ion exchange reaction KO_2_ + Li^+^ → K^+^ + ½ Li_2_O_2_ + ½ O_2_. Figure 3a demonstrates evidently that the morphology of lithium peroxide precipitated after the chemical reaction of KO_2_ with Li^+^ ions is quite similar to that of lithium peroxide produced in Li–O_2_ cells (Figure 2). In the former case the precipitate, which was found to contain Li_2_O_2_ and residual KO_2_ (Figure 3b), exhibited similar crystal clusters composed of thin platelets. This finding suggests that lithium peroxide particles can be formed right upon the formation of superoxide anions without the influence of the surface of the electrode. After being produced by either the electrochemical ORR or the chemical reaction with KO_2_, the superoxide anions associate with Li^+^ which leads to the unstable intermediate that is further converted to Li_2_O_2_. The growth of the lithium peroxide plate-like crystals and their further assembly can already occur in the liquid electrolyte.

**Scheme 1:**
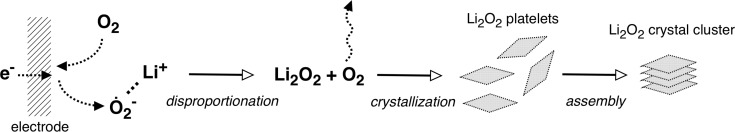
The suggested scheme for the formation of the Li_2_O_2_ precipitate during the discharge of a Li–O_2_ cell.

Scheme 1 illustrates the suggested mechanism of the deposit growth during discharge. At first, molecular oxygen that is dissolved in the electrolyte is reduced to superoxide ions, O_2_ + e → O_2_^−^. These superoxide ions, which carry a negative charge, move away from the electrode surface driven by diffusion [16]. The subsequent disproportionation into a peroxide ion and oxygen, probably by intermolecular collisions as a rate limiting step, O_2_^−^ + O_2_^−^ → O_2_^2−^ + O_2_, results in the generation of lithium peroxide that quickly exceeds a solubility threshold, which was estimated to be about 2.5 mM by (see Figure S3 in Supporting Information File 1). This means that the nucleation proceeds homogeneously and the phase formation is exhausted by the nucleation stage only without an intensive crystal growth because of the relatively high supersaturation that appeared as a result of the fast electrochemical generation of O_2_^−^. After that stage, Li_2_O_2_ platelets seem to be formed as observed recently for carbon electrodes [12]. The generated pristine platelets produce a colloidal system under the conditions of their continuous homogeneous nucleation. Most of the layered colloidal systems tend to aggregate if no sufficient electrostatic or steric stabilization is provided. In the particular case of lithium peroxide platelets, they gradually produce submicron crystal clusters with complex morphology.

Thus this study indicates that Li_2_O_2_ crystal clusters are deposited onto the electrode. This layer, however, remains porous, which allows a further mass transport between the electrode and the electrolyte. These deposits can then lose their electric contact with the electrode and thus additionally limit the rechargeablity of the Li–O_2_ cell. On the other hand, the special morphology of Li_2_O_2_ provides a larger surface compared to well-facetted crystals or uniform films that might allow a faster recharge. This idea becomes highly interesting in view of recent findings of Chen et al. [17] who suggested a liquid phase mediator. Further work on this topic is in progress.

## Supporting Information

File 1 Experimental details.
