# Supplementary material for: Lithium peroxide crystal clusters as a natural growth feature of discharge products in Li–O2 cells
Source: Beilstein J Nanotechnol. 2013 Nov 15;4:758–62. doi: 10.3762/bjnano.4.86 (PMC3869314; doi:10.3762/bjnano.4.86)
Supplement: File 1 — Experimental details. [file Beilstein_J_Nanotechnol-04-758-s001.pdf]

Supporting Information

for

Lithium peroxide crystal clusters as a natural growth feature of discharge products in Li–O_2 cells

Tatiana K. Zakharchenko[†1], Anna Y. Kozmenkova[†1], Daniil M. Itkis*[1,2] and Eugene A. Goodilin[1,2]

Address: [1]Department of Materials Science, Moscow State University, Leninskie gory, Moscow 119991, Russia and [2]Department of Chemistry, Moscow State University, Leninskie gory, Moscow 119991, Russia

* Corresponding author

Email: Daniil M. Itkis* - daniil.itkis@gmail.com

[†] Authors contributed equally to this work.

Experimental details

Preparation of porous gold electrodes

Porous gold electrodes were prepared by etching Au/Ag alloy foils with concentrated nitric acid at room temperature for 24 h. Before etching the foil was roll-pressed to a thickness of 40 µm and annealed at 800 °C for 2 h for mechanical stress relaxation. After etching the mean size of the pores in the electrodes was estimated to be about 20 nm (Figure S1a). A subsequent annealing of the porous gold foils at 300 °C for

1 h resulted in an increase of the mean pore size up to 180–200 nm. The residual amount of silver in the porous gold electrodes was estimated to be lower than 1 atom %.

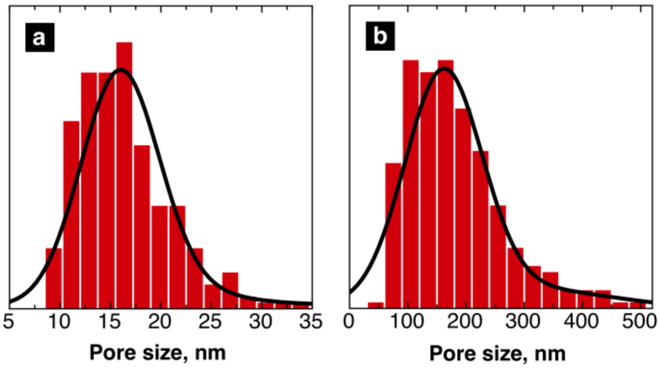

Figure S1: Pore size distribution for porous gold electrodes after (a) etching for 24 h and (b) after etching in nitric acid followed by annealing at 300 °C for 1 h. The pore size distribution was estimated by a statistical analysis of multiple SEM images with approximately 200–250 pores counted in total.

Electrochemical measurements

The discharge of porous gold electrodes was performed in two-chamber/three-electrode electrochemical cells (Mikromasch DC-Combi). Metallic lithium foil (China Energy Lithium) was utilized as counter (CE) and reference electrodes (RE). The lithium electrodes were separated from the porous gold cathode by a solid Li^+-conductive glass–ceramic electrolyte (Ohara inc.). To provide a better contact between the solid ionic conductor, the RE and the CE, a piece of porous polypropylene separator soaked in 1 M solution of $LiClO_4$ (Aldrich, battery grade) in propylene carbonate and 1,2-dimethoxyethane mixture (7:3 vol., Aldrich, anhydrous) was placed in between. The porous gold electrodes were wetted with 1 M solution of LiTFSI (Aldrich) in dimethyl sulfoxide (Aldrich, anhydrous) and placed on top of the solid electrolyte.

The cells were assembled inside an Ar filled glove box (Labconco Protector CA). After assembly, the cathode was flushed with pure oxygen and held at a constant oxygen pressure of about 1 atm during its discharge.

Galvanostatic discharge was performed by using the Biologic SAS MPG2 battery cycler. Current densities were normalized per 1 cm^2 of the electrode geometric area.

Analysis of materials

A field-emission SEM (Leo Supra 50 VP) was utilized to observe the Li_2O_2 morphology. Elemental analysis was carried out using energy dispersive X-ray spectroscopy (Oxford Instruments INCA). TEM images were collected by using a JEOL JEM-2100F/Cs/GIF. Raman scattering spectra were collected with a Renishaw inVia microscope with Ar^+-laser operating at 514 nm wavelength. The reference spectra of pure Li_2O_2, KO_2 and LiTFSI are shown in Figure S2.

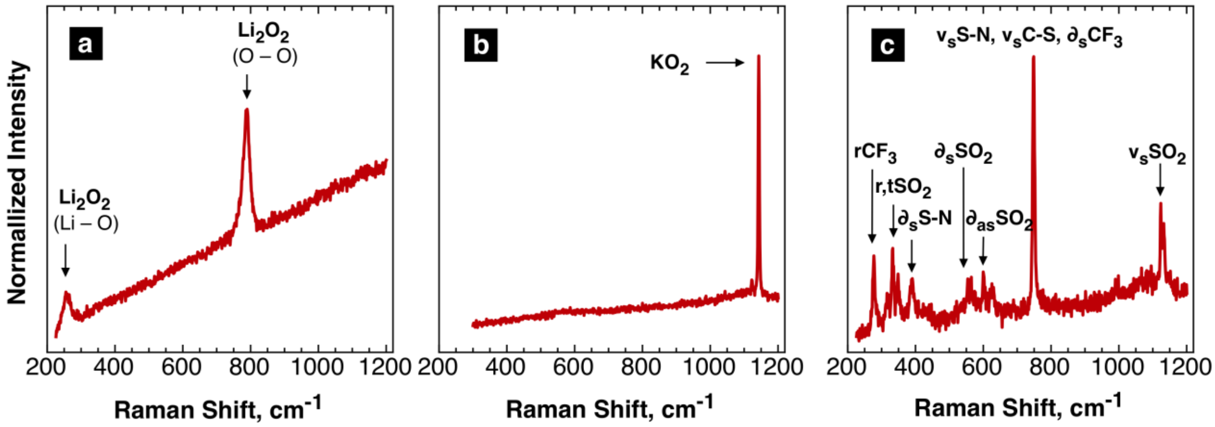

Figure S2: Raman spectra of (a) Li_2O_2, (b) KO_2 and (c) LiTFSI. The symbols have their usual meaning: ν - stretching, ∂ - bending, t - twisting, r – rocking, s and as – symmetric and asymmetric vibrations, respectively.

To estimate the solubility threshold of Li_2O_2 in the electrolyte, ionic-coupled plasma mass-spectroscopy measurements were performed. Lithium peroxide was prepared

as described in Feher et al. (*Chem. Ber.* **1953,** *86,* 1429–1437) using metallic lithium (China Energy Lithium Co.) and ethanol (Aldrich). Li_2O_2 was dried over P_2O_5 in vacuum (10^{-2} mbar) in a desiccator. Thus synthesized lithium peroxide was washed in pure DMSO (Aldrich, anhydrous) three times to remove all possible residuals of impurities (e.g., LiOH) that can be easily dissolved. Then lithium peroxide powder was vigorously stirred in 0.1 M LiTFSI in DMSO. The resulting solution was filtered, 100-fold diluted with deionized water (Millipore Elix) and analyzed using a Perkin Elmer ELAN DRC II. The concentration of Li^+ ions in solution was found to be 0.105 ± 0.001 mol/L. Hence, the increase of the Li concentration after stirring with Li_2O_2 in electrolyte is believed to be 0.005 mol/L and the solubility of Li_2O_2 in 0.1 M solution of LiTFSI in DMSO was estimated to be about 0.0025 mol/L.

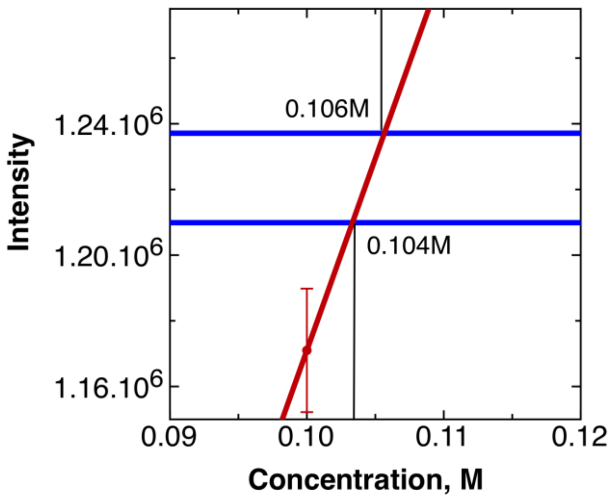

Figure S3: Evaluation of Li concentration in 0.1 M LiTFSI in DMSO after stirring of the latter with Li_2O_2 powder. The red line shows a fragment of the calibration curve, the blue lines limit the range of Li^+ intensities measured for the solution of Li_2O_2 and LiTFSI in DMSO.
